# *Sclerotinia sclerotiorum* growth and aggressiveness are regulated by a mycoviral REP protein

**DOI:** 10.1371/journal.ppat.1013813

**Published:** 2025-12-26

**Authors:** Peihan Shu, Yi-Wen Tseng, Alexander J. Lawrence, Chenchen Feng, Yasi Kiani, Olivia Knopke-Mooney, Rawnaq N. Chowdhury, Danny Lasky, Carol Groves, Damon L. Smith, Kinjal Majumder, Shin-Yi Lee Marzano, Aurélie M. Rakotondrafara

**Affiliations:** 1 Department of Plant Pathology, University of Wisconsin-Madison, Madison, Wisconsin, United States of America; 2 The Ohio State University, Department of Plant Pathology, Toledo, Ohio, United States of America; 3 University of Toledo, Department of Environmental Sciences, Toledo, Ohio, United States of America; 4 Institute for Molecular Virology, University of Wisconsin-Madison, Madison, Wisconsin, United States of America; 5 U.S. Department of Agriculture-Agricultural Research Services, Application Technology Research Unit, Toledo, Ohio, United States of America; Agriculture and Agri-Food Canada, CANADA

## Abstract

The mechanisms through which mycoviruses reduce fungal growth and aggressiveness remain unclear, particularly regarding the viral factors involved and their modes of action. In this work, we investigated the hypovirulence mechanism by which Sclerotinia sclerotiorum hypovirulence-associated DNA virus 1 (SsHADV1), of the species *Gemycircularvirus sclero1*, impaired the necrotrophic plant fungus *Sclerotinia sclerotiorum* growth. We first identified the replication-associated protein (REP) of SsHADV1 as the key factor of hypovirulence. Using different patho-systems, we demonstrated that the viral SsHADV1 REP outside the context of viral infection directly restricts fungal growth and disease development. This slow growth was associated with a reduction in fungal oxalic acid production, which is an essential factor for fungal pathogenicity, and with an increase in fungal susceptibility to sublethal concentration of commercial fungicide. Additionally, the exogenous application of SsHADV1 REP protected sunflower plants from basal stem rot by *S. sclerotiorum*. In tobacco plants, the overexpression of SsHADV1 REP primes plant stress-related and immune responses, likely enhancing the hypovirulence activity against *S. sclerotiorum*. Hypovirulence was replicated using the REP of an unrelated ssDNA virus of *S. sclerotiorum* despite their overall low sequence identity. This conserved function of REP is structurally dependent and requires a functional ATPase domain. This study provides the first molecular insight on the mode of action of an hypovirulent mycovirus. It further establishes the ecological role of mycoviruses and their encoded proteins as potential drivers of fungal disease outcome and severity.

## Author summary

Hypovirulent mycoviruses offer a promising potential for developing sustainable bio-fungicides. We identified the replication-associated protein (REP) of Sclerotinia sclerotiorum hypovirulence-associated DNA virus 1 (SsHADV1) as a natural anti-fungal agent against economically significant fungal plant pathogens, suppressing its growth and aggressiveness. To reveal its biological function, the non-pathogenic, gram-positive rhizobacterium *Bacillus subtilis* was engineered to serve as a delivery platform for SsHADV1 REP. Notably, pre-treating sunflower plants with culture of REP-secreting bacteria prevented root rot infection by *Sclerotinia sclerotiorum*. Furthermore, the application increased effectiveness of low dose of boscalid fungicide on the fungus. Such function was replicated with the REP of an unrelated ssDNA virus of S. *sclerotiorum* that shares similar structure and with a functional ATPase domain. This work highlights the molecular effect of mycoviruses on the plant-fungus interaction and their potential ability to regulate disease outcome.

## Introduction

Intensive monoculture practices and chemical use have spurred the evolution of pathogens with stronger virulence and pesticide resistance. Of particular concern are the plant fungal pathogens that can cause extensive crop losses between 10–23% every year [1]. Some fungi produce large numbers of airborne spores that can spread rapidly to susceptible crops. Some of these same fungi can also produce survival structures that persist in the soil for years. Of such concern is *Sclerotinia sclerotiorum* (Lib.) deBary, a hemibiotroph with a strong necrotrophic phase that can cause diseases such as Sclerotinia stem rot and white mold [2]. *S. sclerotiorum* has a broad host range infecting more than 400 plant species, including many economically significant crops [3]. It forms sclerotia, which are dark hard structures that enable it to survive harsh conditions and overwinter in the soil for several years [4]. Due to the limited availability of resistant plant varieties, management of *S. sclerotiorum* in some crops relies heavily on fungicides, increasing the propensity for development of fungicide-resistance, and can bring negative impact to the environment [5,6]. As a result, there is an escalating demand for innovative antifungal control methods that are both sustainable and effective.

Mycoviruses or fungal viruses, are found in nearly all fungal taxa, including those that cause serious diseases in economically or agriculturally important plants [7]. While the majority of mycoviruses do not have detrimental impact on their hosts, a small fraction have evolved distinct strategies to reduce the vegetative growth and disease severity of their fungal hosts, thus gaining significant attention as a promising biocontrol strategy [8]. Cryphonectria hypovirus 1 (CHV1) has been successfully used to manage chestnut blight caused by *Cryphonectria parasitica* in chestnut forests in Europe [9]. In *S. sclerotiorum*, the most extensively studied mycovirus with hypovirulence properties is Sclerotinia sclerotiorum hypovirulence-associated DNA virus 1 (SsHADV1), of the species *Gemycircularvirus sclero1*. SsHADV1 is a circular, single-stranded DNA virus with a genome of approximately 2 kb in length, which encodes only two proteins: the replication-associated protein (REP), consisting of around 330 amino acids and is essential for viral replication within infected cells, and the capsid protein (CP) [10]. The functions of mycovirus proteins in the *Genomoviridae* family remain largely unknown. In closely related plant DNA geminiviruses, REP is a multi-functional protein involved in several processes including controlling host cell cycle and DNA replication in the infected cells [11]. SsHADV1 has been reported to transform its host fungus into an endophyte and to change expression patterns of the infected plant including activating plant immunity [10,12,13]. Transcriptome analysis revealed that SsHADV1 infection suppresses the expression of fungal antiviral RNA silencing and virulence factor-related genes [14]. Application to plants of purified viral particles [15] or hyphal fragments containing SsHADV1 particles [12] protected plants against *S. sclerotiorum* infection and damage.

In this study, using different patho-systems, we provided the first insight on the mechanism through which SsHADV1 regulates fungal pathogen aggressiveness at least in a laboratory setting, isolating the viral factor involved and establishing their molecular effect on the plant-fungus interaction.

## Results

### Stable expression of SsHADV1 REP in *Sclerotinia sclerotiorum* inhibits fungal growth

**Fig 1:**
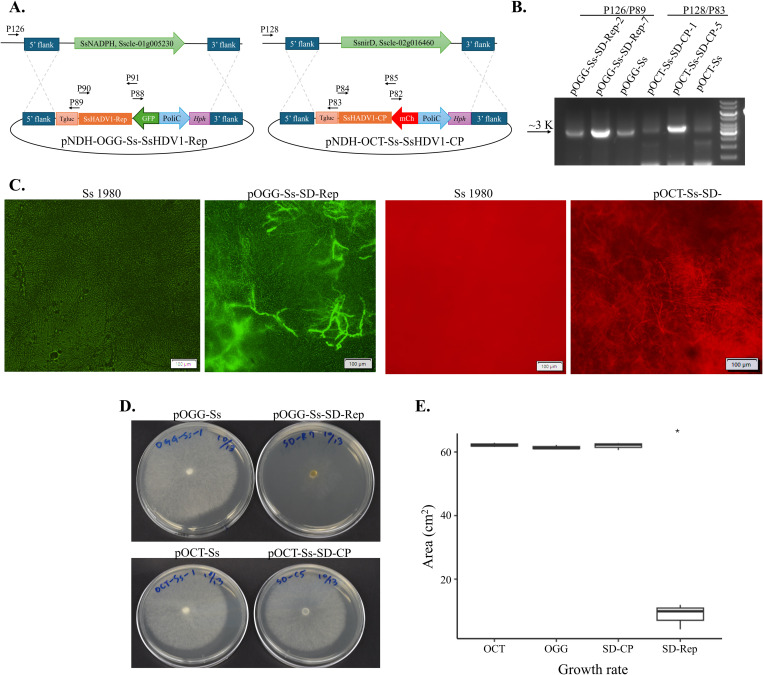
Stable expression of the viral proteins, SsHADV1-Rep and -CP in *S. sclerotiorum* and the fungal growth rate assay. **(A)** The construct maps of the site-direct mutagenesis vectors with SsHADV1 viral proteins for *S. sclerotiorum* transformation. The vectors, pOGG-Ss-SD-Rep and pOCT-Ss-SD-CP, were constructed by using the In-fusion Snap assembly master mix. **(B)** Confirmation of the 5’ region of homologous integration transformants by using the PCR primers (P126/P89 and P128/P83) (S4 Table). Gene displacement was validated for each transformant. **(C)** Fluorescence microscopy images showing the stable expression of the fused green fluorescent and mCherry fluorescent proteins of *S. sclerotiorum* transformants compared to the wild-type *S. sclerotiorum* 1980. Scale bar = 100 μm. **(D, E)** Growth comparison between the transformants and the empty vector control with statistics to the right, showing that SsHADV1-Rep expressing transformant had a significantly reduced growth (*P* < 0.05) and sparsely distributed hyphae on PDA.

**Fig 2:**
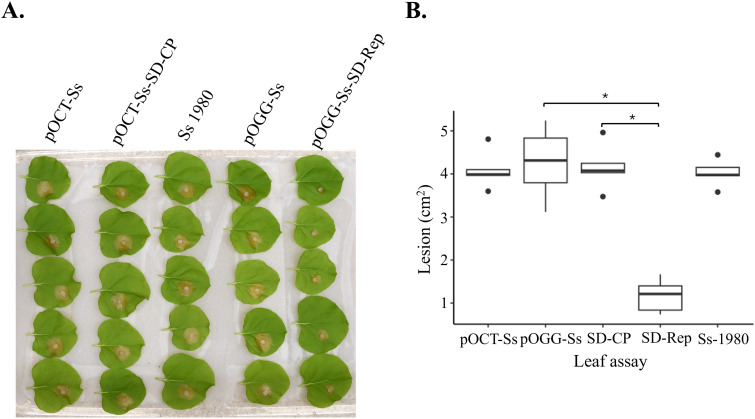
pOGG-Ss-SD-Rep transformant exhibited significantly reduced lesion size and virulence. **(A, B)** The detached *N. benthamiana* inoculation leaf assay with the different *S. sclerotiorum* transformants and the untransformed *S. sclerotiorum* Ss1980 culture. The 0.5-cm-diameter mycelial plugs were placed with mycelium-side down on the leaf surface. Images of the lesion were captured 24 hr post inoculation and the lesion diameters (cm^2^) were measured. The results showed significantly reduced virulence caused by SsHADV1-Rep (p < 0.05) compared to the virus-free and other transformants, including empty vectors and SsHADV1-CP. Ss 1980: untransformed Ss; pOCT-Ss: Ss transformed with pOCT empty vector; pOCR Ss-SD-REP: REP transformant; pOGG-Ss: Ss transformed with pOGG empty vector; POGG-Ss-CP: CP transformant.

To isolate the hypovirulence factor of SsHADV1, the SsHADV1 encoded genes including the replication-associated protein (REP) and the coat protein (CP) genes were each fused to a fluorescent reporter gene and were site-specifically integrated via homologous recombination into virus-free *S. sclerotiorum* strain 1980 to replace the nitrate reductase *NADPH* and nitrite reductase *nirD* fungal genes, respectively, which are dispensable for virulence [16]. The SsHADV1 REP gene was fused to a green fluorescent protein (GFP) gene in the pOGG-Ss vector and the CP gene to a mCherry protein gene in the pOCT-Ss vector (Fig 1A). This resulted in the heterologous expression of the two SsHADV1 proteins in *S. sclerotiorum* transformants, which were designated OGG-Ss-SD-Rep and OCT-Ss-SD-CP respectively. Homologous recombination of *S. sclerotiorum* transformants were confirmed by diagnostic PCR [16] for the 5’ and 3’ flanking regions designed for double cross-over to achieve site-directed mutagenesis as intended (Fig 1B). The expression of the fused proteins was next validated with the fluorescence detection of the GFP and mCherry proteins in the OGG-Ss-SD-Rep and OCT-Ss-SD-CP transformed *S. sclerotiorum,* respectively (Fig 1C). When grown on plates, the transformant expressing REP displayed a severely reduced growth rate and sparsely distributed hyphae compared to the empty vector control (*P* < 0.05) (Fig 1D and 1E). By contrast, the transformant expressing CP displayed no significant growth difference compared to the empty vector transformant (Fig 1D and 1E). Note that the empty vector transformants showed similar growth rate (Fig 1D and 1E). When inoculated on *Nicotiana benthamiana* leaves, the OGG-Ss-SD-Rep transformed *S. sclerotiorum* showed limited infection within 24 hours post inoculation (Fig 2). It exhibited significantly reduced lesion size, which is characteristic of hypovirulence when compared to the untransformed *S. sclerotiorum*, the empty vectors and the CP transformants (p < 0.05) (Fig 2).

**Fig 3:**
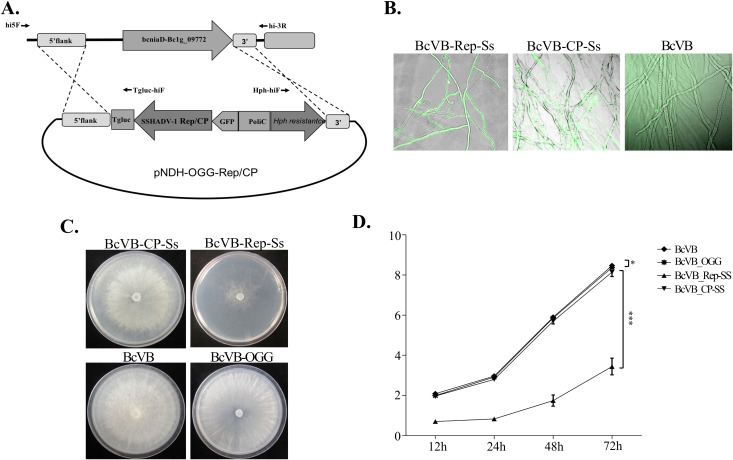
Confirmation of Site-Directed Expression of REP and CP in *B. cinerea.* **(A)** Overexpression scheme by homologous recombination at the 5’- and 3’-flanking regions of a non-essential gene. **(B)** Fluorescence microscopy images showing the stable expression of the fused REP and CP fluorescent proteins of *B. cinerea* transformants compared to the wild-type *B. cinerea.* Scale bar = 100 μm. **(C)** Growth comparison between the transformants and the empty vector control with statistics to the right, showing that REP-expressing transformant had a significantly reduced growth on PDA. BcVB, wild-type *B. cinerea*; BcVB-OGG, BcVB transformed by empty OGG vector; BcVB-CP-Ss, CP transformant; BcVB-REP-Ss, REP transformant.

**Fig 4:**
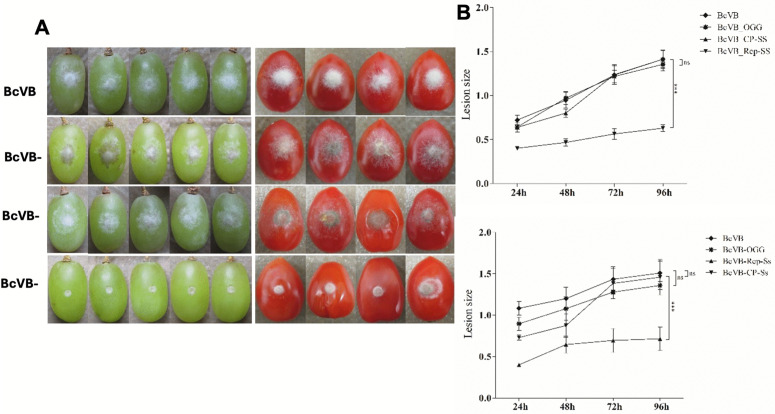
BcVB-REP-Ss Transformant Exhibited Significantly Reduced Growth and Lesion Size. **(A)** Comparison of virulence determinant of SsHADV1-encoding genes by BcVB-CP-Ss and BcVB-REP-Ss transformants on susceptible plants, grapes and tomatoes. Lesion diameters were compared on green grapes and tomatoes inoculated with different transformants. **(B)** Average lesion diameters (cm) measured on 8 fruits after 24, 48, 72, and 96 h after inoculation with the strains from one of the three trials. All three trials showed consistently reduced virulence caused by BcVB-REP-Ss (*P* < 0.05). Upper: grapes; lower: tomatoes.

Similar phenotype of hypovirulence was observed when the SsHADV1 REP was expressed in *Botrytis cinerea*, a closely related fungus to *S. Sclerotiorum* and a non-natural host for SsHADV1 [15] (Fig 3A). The SsHADV1 REP and CP genes were fused to the GFP gene and site-specifically integrated via homologous recombination to replace a non-essential *bcniaD* fungal gene. Homologous recombination was confirmed by diagnostic PCR (S1A Fig). Using RT-PCR, we validated the accumulation of the transcripts with the viral CP and REP genes of about 1 kb in length each from the corresponding *B. cinerea* transformants (S1B Fig). The expression of the GFP was observed in each of the transformants (Fig 3B). When grown on plate, the *B. cinerea* transformant expressing REP (BcVB-REP-Ss) displayed also a severely reduced growth phenotype and sparsely distributed hyphae, as depicted in Fig 3C, when compared to the wild-type *B. cinerea* and the fungal transformant with an empty vector. By contrast, there was no discernible difference in the morphology of BcVB-CP-Ss, the transformant that expresses the SsHADV1 CP protein, from the controls (Fig 3C). When inoculated in tomato and grapes, the BcVB-REP-Ss fungal transformant showed limited infection (Fig 4). It exhibited significantly reduced growth and lesion size (*P* < 0.05) when compared to the wild type and the CP transformants (Fig 4).

Altogether, the results are pointing to the SsHADV1 REP as the potential contributor in hypovirulence.

### Ectopic application of SsHADV-1 REP is sufficient to reduce fungal growth and aggressiveness

**Fig 5:**
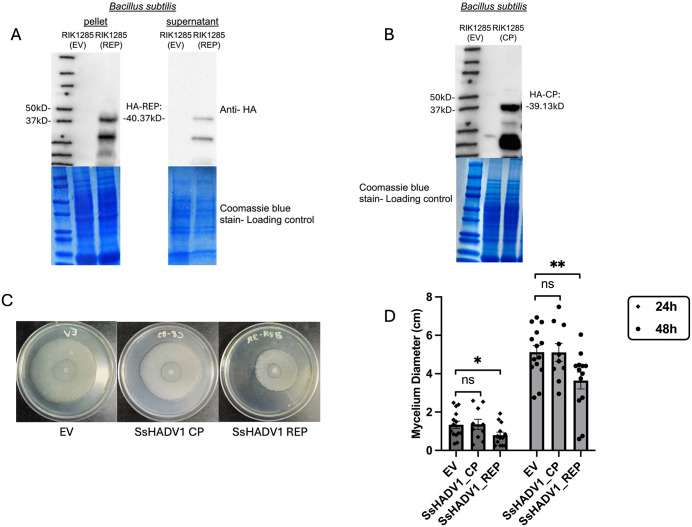
Exogenous Application of the Cell Filtrate Containing SsHADV1 REP Reduced *S. sclerotiorum* Growth and Pathogenicity. **(A)** Detection of SsHADV1 REP expression and secretion by engineered *B. subtilis*. A western blot using an anti-HA tag antibody confirmed the presence of the HA-REP fusion proteins in both bacterial pellet and supernatant of transgenic *B. subtilis* RIK1285(REP). **(B)** A western blot using an anti-HA tag antibody confirmed the presence of the HA-CP fusion proteins in bacterial culture of transgenic *B. subtilis* RIK1285(CP). **(C)** Equal volumes of the *B. subtilis* cell filtrate were added to PDA plates containing spectinomycin, followed by the addition of an *S. sclerotiorum* agar plug. Images of the mycelium were captured at 48 hours post-infection (hpi). **(D)** statistical analysis of the mycelium diameter at 24 and 48 hpi is presented. Error bars represent the SEM (n = 12). Statistical significance was determined using ANOVA followed by Tukey’s HSD test, with significance groups indicated by letters.

To further explore the hypovirulence activity of SsHADV1 REP, we utilized the *B. subtilis* protein expression and secretion system to produce the viral proteins. We transformed HA-tagged SsHADV1 REP and SsHADV1 CP pBE-S vectors into *B. subtilis* RIK1285. Western blot analysis validated the expression of the SsHADV1 proteins in *B. subtilis* and secretion into the culture media (Fig 5A and 5B). We next recovered and filtered bacterial culture supernatants from RIK1285(REP), RIK1285(CP) and empty vector RIK1285(EV) transformed bacteria and applied the culture filtrates directly to fungal mycelium growing on nutrient media. The exogenous application of the SsHADV1 REP-containing culture filtrate onto actively growing *S. sclerotiorum* slowed down fungal growth within 48 hours, while the filtrate containing the SsHADV1 CP showed no effect, like the EV control treatment, in line with the hypovirulence activity of the SsHADV1 REP (Figs 5C and 4D).

**Fig 6:**
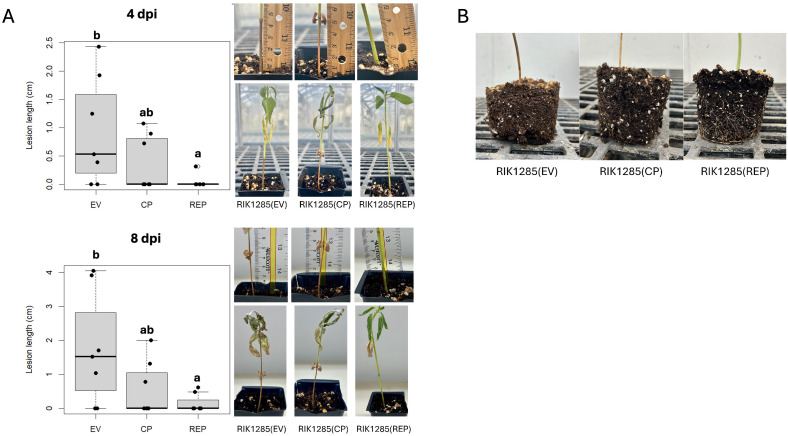
Application of RIK1285(REP) Protects Sunflowers from White Mold. Sunflower plants were treated with equal amounts of bacterial suspensions containing RIK1285(EV), RIK1285(CP) and RIK1285(REP) by soaking the soil. Two days after the initial treatment, an additional 10 ml of bacterial suspension was applied, followed by inoculation of the root mass with an *S. sclerotiorum* agar plug. **(A)** Analyzed lesion length and photographs of treated sunflowers at 4 and 8 days post-fungal infection (dpi). Statistical significance (n = 8) was determined using ANOVA followed by Tukey’s HSD test, with significance groups indicated by letters. **(B)** Representative images of treated sunflower roots at 8 dpi.

To support the disease suppression activity of SsHADV1 REP, six-week-old V6 stage sunflower plants were root-irrigated with RIK1285(REP), RIK1285(CP), and RIK1285(EV) *B. subtilis* suspension cultures. Four days post-treatment, plants were root-inoculated with actively growing *S. sclerotiorum* mycelium. The development of basal stem rot was assessed on day 4 and day 8 post *S. sclerotiorum* inoculation [17]. We first confirmed that similar bacterial colony forming units were present in the soil across all treatments for the duration of the experiment (S2 Fig). Remarkably, by day 4, plants treated with RIK1285(REP) showed no signs of stem rot comparing to the control treatments, which already exhibited an average stem lesion length of 0.93 cm (*P* < 0.05). By day 8, most plants treated with RIK1285(REP) exhibited little sign of collapse from basal stem rot (S1 Table). The observed lesions along the stem were much smaller compared to the RIK1285(EV) control group at 8 days (*P* < 0.05) post-inoculation (Fig 6A). Additionally, the REP-treated sunflowers showed healthier root systems when compared to the EV control treatment (Fig 6B). We noted that bacteria secreting SsHADV1 CP seem to provide some level of protection to the plants when compared the EV-control, but not to the extent of the REP treatment. Altogether, the findings are in line with the hypovirulence activity of SsHADV1 REP.

### SsHADV1 REP reduces oxalic acid production, a key fungal pathogenicity factor, and enhances fungal susceptibility to sublethal fungicide concentrations

**Fig 7:**
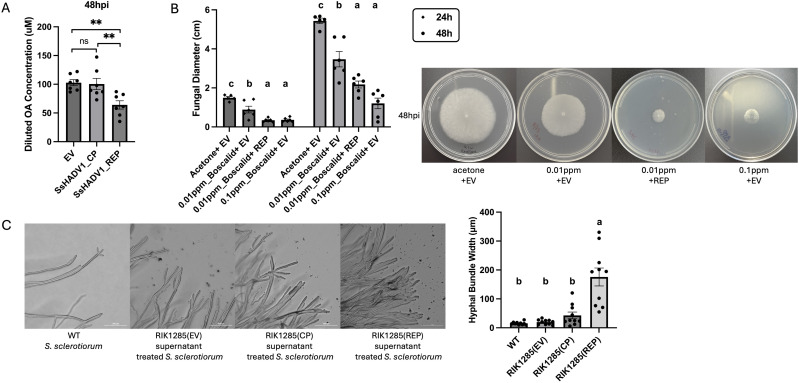
SsHADV1 REP Reduces Oxalic Acid Production and Enhances Fungal Susceptibility to Sublethal Fungicide Concentrations. **(A)** A standard plate assay was performed. At 48 hpi, three same-sized agar plugs from each treatment were collected. Each plug was placed in 500 µL of nuclease-free water and the solution was then collected as a sample for analysis using the Oxalate Assay Kit. The estimated Oxalic Acid level of each treatment is presented. Error bars represent the SEM (n = 7). Statistical significance analyzed using a one-tailed t-test, is indicated as follows: “*” for *P* < 0.05 and “**” for *P* < 0.01. **(B)** A plate assay was conducted with *S. sclerotiorum* 1980 strain using PDA plates amended with boscalid at final concentrations of 0.1 ppm and 0.01 ppm. PDA medium with 0.01% (vol/vol) acetone was used as a control. Statistical analysis of mycelium diameter at 24 and 48 hours, along with photographs of the plates taken at 48 hours, are presented. Statistical significance (n = 6) was determined using ANOVA followed by Tukey’s HSD test, with significance groups indicated by letters. **(C)** Representative images under light microscope of *S. sclerotiorum* 1980 hyphae at 48 hpi treated with the supernatants of RIK1285(EV), RIK1285(CP), and RIK1285(REP), and the measurement of the hyphae width. Healthy wild-type *S. sclerotiorum* serves as the control. Scale bar = 300 μm. Hyphal bundle width was measured using ImageJ software. For each plate, three regions were randomly selected, and measurements were taken based on the scale bar provided in the image (n = 10).

The observed slow fungal growth phenotype on plates was associated with a significantly lower level of oxalic acid production, which is an essential factor for fungal infection [18], from mycelium 48 hours following SsHADV1-REP treatment (*P* < 0.01), when compared to the SsHADV1-CP-treated or EV-treated *S. sclerotiorum* (Fig 7A). Oxalic acid levels were quantified from culture agar plugs using the Oxalate Assay Kit (Sigma-Aldrich) following the manufacturer’s instructions. This reduction in oxalic acid production correlated with a decreased expression of the oxalic acid biosynthesis related gene *oah1* mRNA (*P* < 0.05) (S3 Fig), in line with previous study reporting that SsHADV1 fungal infection suppressed the expression of virulence factor related genes [14].

We next assessed whether the application of REP would increase fungal susceptibility to chemical Boscalid fungicide, normally used to control *S. sclerotiorum* [19]. Through serial dilution of the fungicide and fungal plate assays, we first determined the sub-lethal concentration of the fungicide that resulted in a slight reduction in *S. sclerotiorum* growth when compared to acetone treated control and that was below the manufacturer’s indicated the lethal concentration LC50 of 0.1 ppm. This concentration was set at 0.01 ppm. Next, culture filtrates of RIK1285(REP) and RIK1285(EV) were applied to PDA plates containing 0.01 ppm boscalid and an actively growing *S. sclerotiorum* agar plug. The 0.01 ppm fungicide alone caused a slight reduction in fungal growth within 48 hours compared to the acetone buffer control *(P < *0.01). However, when this sub-lethal concentration of fungicide was combined with the RIK1285(REP) cell filtrate, a strong reduction in fungal growth was observed compared to the 0.01 ppm fungicide (*P* < 0.05), resulting in growth inhibition to similar extend as the LC50 treatment at 0.1 ppm (*P* > 0.05) (Fig 7B).

This increased fungal susceptibility to fungicide prompted us to test whether the REP treatment compromised the fungal membrane permeability, as previously reported for some anti-fungal defensin peptides [20]. We thus used the SYTOX Green uptake assay as an indicator of membrane integrity. The SYTOX Green renders cells with compromised plasma membrane to fluoresce bright green. We found that unlike the UV-treated control hyphae that turned green fluorescent, *S. sclerotiorum* cells treated with SsHADV1 REP did not uptake SYTOX Green, similarly to the wild-type and empty vector controls, suggesting that REP did not affect membrane permeabilization (S4 Fig). However, the observation of the fungal hyphae under the microscope revealed the aggregation of the parallel-oriented hyphae in thicker bundles, with a width up to 180 μm, upon exposure to the REP when compared to the non-treated and EV control and the CP treatments, in which the hyphae remained largely separated, with an average hyphae width of about 20 μm (Fig 7C). The formation of hyphae bundles was previously reported as a response to environmental stress [21].

### An intact ATPase domain of the SsHADV1 REP is important for its hypovirulence function

**Fig 8:**
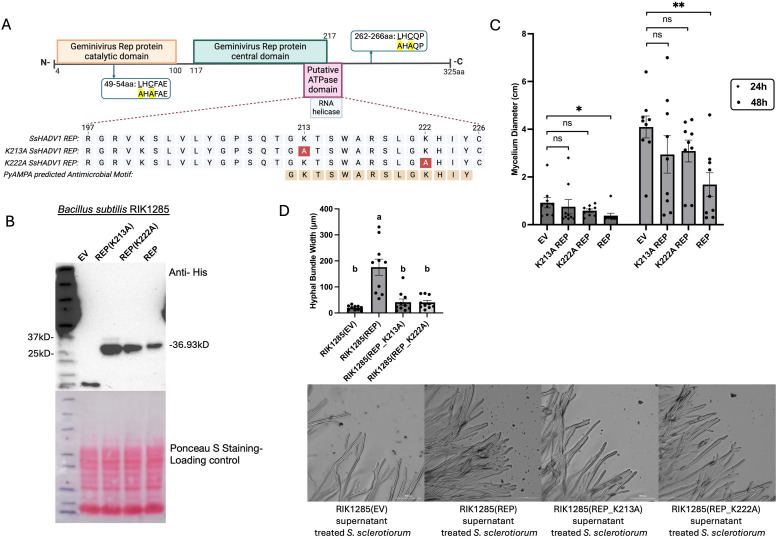
Mutation in the SsHADV1 ATPase domain results in a loss of hypovirulence function. **(A)** SsHADV1 REP sequence with the putative LXCXE motifs and the ATPase domain predicted by the Pfam motif library and the antimicrobial motif predicted by PyAMPA highlighted, and the corresponding mutant sequences. Figure created with BioRender (Shu, P. (2025) https://BioRender.com/uj9mzcf) **(B)** A Western blot using an anti-His tag antibody confirmed the presence of the REP-His fusion proteins in transgenic *B. subtilis* RIK1285(REP), RIK1285(REP-K213A) and RIK1285(REP-K222A). **(C)** Equal volumes of the *B. subtilis* cell filtrates were added to PDA plates containing spectinomycin, followed by the addition of an *S. sclerotiorum* agar plug. Statistical analysis of the mycelium diameter at 24 and 48hpi is presented. Error bars represent the SEM (n = 9). Statistical significance analyzed using a one-tailed t-test, is indicated as follows: “*” for *P* < 0.05 and “**” for *P* < 0.01. **(D)** Representative images under light microscope observation of *S. sclerotiorum* 1980 hyphae at 48 hpi treated with the supernatants of RIK1285(EV), RIK1285(REP), RIK1285(REP_K213A), and RIK1285(REP_K222A). Scale bar = 300 μm. Hyphal bundle width was measured using ImageJ software. For each plate, three regions were randomly selected, and measurements were taken based on the scale bar provided in the image (n = 10).

A conserved function of REP in ssDNA plant geminiviruses is the reprogramming of the cell cycle to favor viral replication, which is dependent upon a conserved LXCXE motif [22,23]. We then first identified the potential LXCXE motif in SsHADV1 REP sequence. Our analysis revealed two putative LXCXXE (**L**H**C**FA**E**) and LXCXX (**L**G**C**QP) motifs at position 49–54 and 262–266 (Fig 8A). We next mutated the motifs into AHAFAE and AGAQP motifs respectively and tested the mutant REPs ability to control *S. sclerotinia* growth in our standard plate assay. The assay revealed that these putative LXCXE mutant REPs behaved similarly to the wild-type sequence in limiting fungal growth, suggesting that these motifs may not be relevant for hypovirulence (S5 Fig).

Next, the use of PyAMPA, a computational tool for prediction of antimicrobial motifs [24], predicted a 14-amino acid motif “GKTSWARSLGKHIY” within the SsHADV1 REP sequence to have anti-microbial property (S2 Table). This motif encompasses the putative SsHADV1 REP ATPase domain at position 205–226 [25] (Fig 8A). To test the relevance of this motif in hypovirulence, we individually mutated the two lysines (K) at position 213 and 222 within the predicted peptide motif into alanine (A), which would largely neutralize the positively charged surface area. We then expressed each mutant REPs using the *B. subtilis* system for which the expressions were validated by Western blot (Fig 8B). The plate assay results showed a loss of function. *S. sclerotiorum* displayed limited fungal growth slow-down within 48 hours when treated with the SsHADV1 REP mutants (REP K213A and REP K222A) and compared to the wild-type sequence (*P* > 0.05) (Fig 8C). Additionally, microscopy observation revealed that the treatment with the K mutant REPs did not induce the bundling of the fungal hyphae as observed following treatment with the wild-type REP sequence (Fig 8D). The hyphae remained largely separated similarly to the control. Altogether, the finding reveals that the motif within the ATPase domain is likely needed for the REP hypovirulence activity.

**Fig 9:**
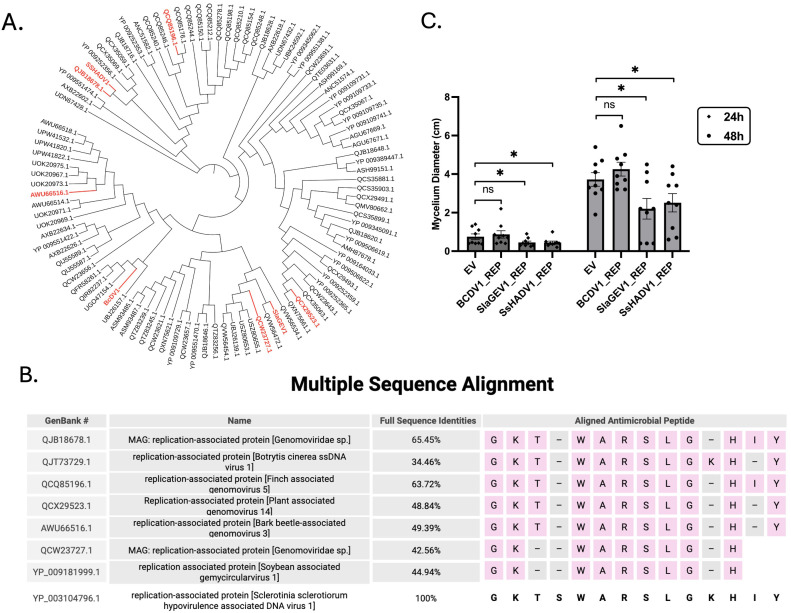
Phylogenetic and Sequence Analysis Reveal Conserved Antimicrobial Motif Among Mycoviral REP Protein Clusters. **(A)** Phylogenetic tree of 98 REP sequences of DNA viruses obtained from metagenomic analysis [26] generated using IQ-TREE, with branch lengths omitted. SsHADV1 REP, SlaGEV1 REP, BCDV1 REP, and other representative REPs from each synthesized cluster are highlighted. **(B)** Alignment of the SsHADV1 REP antimicrobial peptide region across selected mycovirus-like REP sequences. Figure created with BioRender (Shu, P. (2025) https://BioRender.com/epui2wd). **(C)** Equal volumes of the *B. subtilis* cell filtrates were added to PDA plates containing spectinomycin, followed by the addition of an *S. sclerotiorum* agar plug. Statistical analysis of the mycelium diameter at 24 and 48hpi is presented. Error bars represent the SEM (n = 9). Statistical significance analyzed using a one-tailed t-test, is indicated as follows: “*” for *P* < 0.05.

To explore the potential sequence conservation of the predicted anti-microbial peptide among REP proteins, we first performed an alignment of 98 REP sequences of DNA viruses obtained from metagenomic analysis [26] and identified five different clusters. This phylogenetic tree was generated with IQTREE, with branch lengths ignored (Fig 9A). Next we selected representative sequences from each cluster and observed that the predicted 14-amino acid anti-microbial peptide is overall conserved among the different REPs despite their overall low sequence identity to the SsHADV1 REP (Fig 9B). To show potential function conservation, two unrelated REP proteins were tested for their hypovirulence activity. These included the REP from *Soybean leaf-associated gemycircularvirus-1* (SlaGemV1) [27] a mycovirus of *S. sclerotiorum* with a 45% sequence identity to SsHADV1 REP, and that bears 9 out of the 14 amino acids from the predicted antimicrobial motif “GK—WARSLG-H--” (dashed are the unmatched sequences), and the REP [28] from *Botrytis cinerea ssDNA virus* (BcDV1), a mycovirus of *B. cinerea* [29], with an overall 34% sequence identity to SsHADV1 REP and with 12 out of the 14 amino acids from the predicted antimicrobial motif “GKT-WARSLGKH-Y”. The plate assay showed that the SlaGemV1 REP protein limited *S. sclerotiorum* fungal growth to similar extent as the SsHADV1 REP, in line with the hypovirulence activity, but the BcDV1 REP failed to do so (Fig 9C). Altogether, the findings revealed that the motif within the ATPase sequence of REP is important but likely not sufficient for hypovirulence.

**Fig 10:**
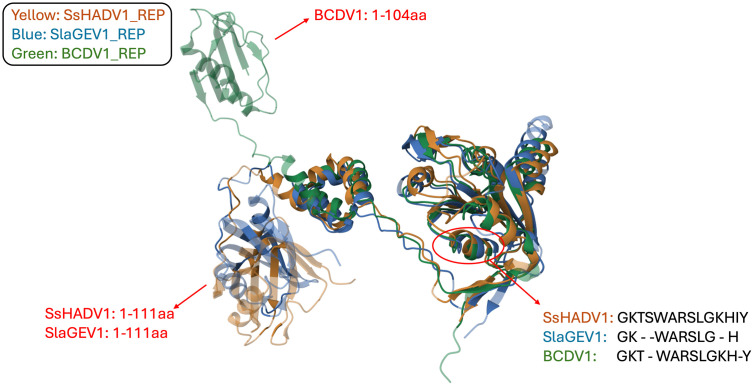
Structural alignment of REP proteins. The SsHADV1, SlaGemV1 and BcDV1 REP structures were predicted using AlphaFold2 via COSMIC2 [30], and the resulting PDB files were visualized using RCSB [31]. SsHADV1 REP is shown in yellow, SlaGemV1 REP in blue, and BCDV1 REP in green. Arrows indicate the regions with structural similarities and differences among the REP. The red circle highlights the overlapping predicted 14 amino acid antimicrobial motif region.

The low amino acid sequence identity between the SsHADV1 and SlaGemV1 REP proteins that showed hypovirulence activity prompted us to investigate whether structural similarity might be consistent with the shared functional role. We thus produced a structural alignment of REP 3D models using AlphaFold2 [30,31]. The results revealed the striking structural similarities of the SsHADV1, SlaGemV1 and BcDV1 REP proteins including the region encompassing the predicted anti-microbial motif (Fig 10). However, the BcDV1 REP, which does not have hypovirulence activity, displayed a different folding of its N-terminal (position 1–104) when comparing to the two other REPs. This leads us to conclude that the overall structural conservation between the SsHADV1 and SlaGemV1 REP is likely consistent with the potential shared mechanism in hypovirulence.

### SsHADV1 REP transient expression in plants inhibits fungal growth

**Fig 11:**
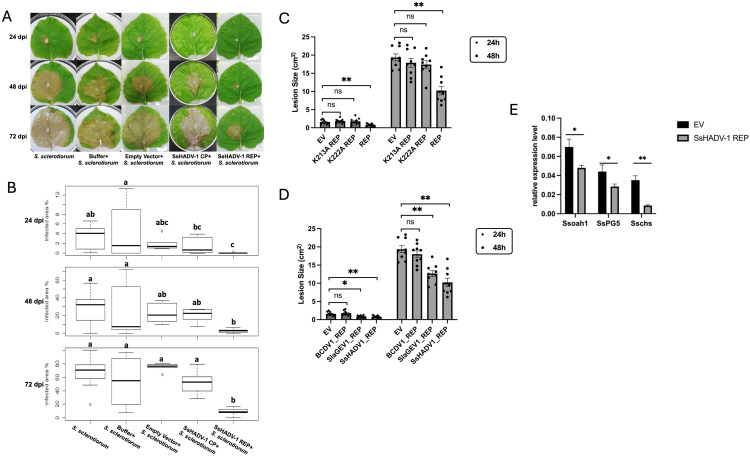
Transient Expression of SsHADV1 REP in Plants Significantly Reduces *S. sclerotiorum* Virulence and Confirms the Role of the ATPase Domain and Conservation of Hypovirulence Function. *N. benthamiana* leaves were infiltrated with MES buffer and agrobacteria GV3101 expressing either an empty vector (EV) control, SsHADV1 CP, or SsHADV1 REP. At 48 hours post-infiltration, the leaves were exposed to a plug of actively growing *S. sclerotiorum* (1980). **(A)** The extent of leaf necrosis was recorded up to 72 hours post-infection (hpi); **(B)** Boxplot showing lesion area measurements at 24-, 48-, and 72hpi. Statistical significance (n = 9) was determined using ANOVA followed by Tukey’s HSD test, with significance groups indicated by letters; **(C)** *N. benthamiana* leaves were infiltrated with agrobacteria (OD600 = 0.8) expressing SsHADV-1 REP, empty vector (EV) control, and two ATPase REP mutants, **(D)** and with the unrelated SlaGEV1 REP and BCDV1 REP. 48 hours post- infiltration, the leaves were exposed to WT *S. sclerotiorum* (1980) plug. Leaf necrosis was recorded up to 48 hpi, and statistical analysis of the lesion area at 24 and 48 hpi is shown. Error bars represent the SEM. Statistical significance analyzed using a one-tailed t-test, is indicated as follows: “*” for *P* < 0.05 and “**” for *P* < 0.01. **(E) (C)** Leaf tissue from both necrotic and healthy areas was collected for total RNA extraction at 48 hours post-fungal infection, followed by RT-qPCR analysis using the housekeeping gene *β-tubulin* and *elongation factor 1-alpha (EF1a).* The relative expression of *S. sclerotiorum* pathogenicity and growth-related genes is shown. Error bars represent the standard error of the mean (SEM), based on three replicates per treatment. Statistical significance analyzed using a one-tailed t-test, is indicated as follows: “*” for *P* < 0.05 and “**” for *P* < 0.01.

To gain additional insight on the effect of SsHADV1 REP on the plant-fungus interaction and on disease outcome, the viral proteins were transiently expressed in *Nicotiana benthamiana* plants via agro-infiltration followed by fungal inoculation. The over-expression of the SsHADV1 REP protein limited necrotic damage by *S. sclerotiorum* with significant reduction of the size of the lesion (*P* < 0.05) within 72 hours post inoculation, when compared to that of the SsHADV1 CP and the control groups, which included the non-treated and mock infiltration of *N. benthamiana* leaves with buffer or empty vector (EV) (Fig 11A and 11B). This is in line with the ability of SsHADV1 REP to regulate fungal aggressiveness. A reverse phenotype was reproduced with the transient expression of the ATPase K mutant REPs. The overexpression of the K213A and K222A SsHADV1 REP mutants in *N. Benthamiana* leaves showed a limited control of *S. sclerotiorum* disease progression when compared to the wild type SsHADV1 REP sequence at 48 hours post inoculation, in line with the relevance of an intact ATPase domain for function (Fig 11C). Furthermore, when we transiently expressed the SlaGemV and the BcDV1 REPs in *N. benthamiana* leaves followed by fungal inoculation, the SlaGemV REP but not the BcDV1 REP efficiently slowed down *S. sclerotiorum* growth (Fig 11D), reproducing the phenotype previously observed on plates (Fig 9C).

**Fig 12:**
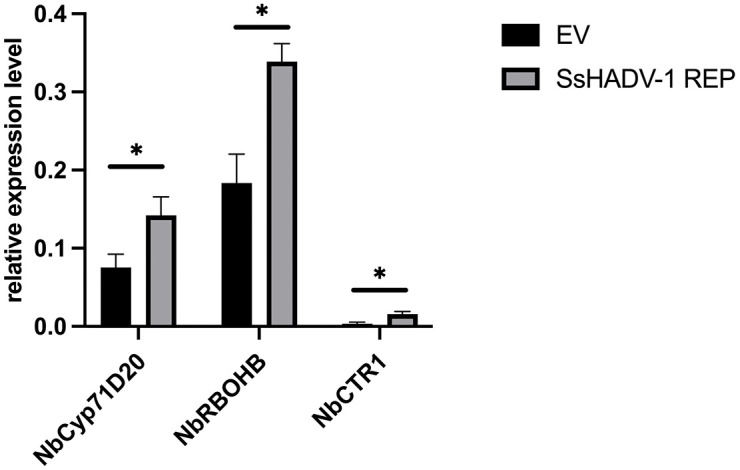
*N. benthamiana* Gene Expression Regulated by SsHADV1 REP Transient Expression. Leaf tissue from both necrotic and healthy areas was collected for total RNA extraction at 48 hours post-fungal infection, followed by RT-qPCR analysis using the housekeeping gene *β-tubulin* and *elongation factor 1-alpha (EF1a).* The relative expression of *N. benthamiana* PRR genes is shown. Error bars represent the standard error of the mean (SEM), based on three replicates per treatment. Statistical significance analyzed using a one-tailed t-test, is indicated as follows: “*” for *P* < 0.05 and “**” for *P* < 0.01.

The RT-qPCR analysis of total RNA extracted from the *S. sclerotiorum* inoculated plant tissues revealed that the disease repression by SsHADV1 REP was associated with several fungal factors critical for *S. sclerotiorum* pathogenicity, including the oxalic acid biosynthesis related gene *oah1*, and cell wall-degrading enzyme (*PG5*), as well as growth-related factor such as chitin synthase (*csh*), being downregulated in plants overexpressing SsHADV1 REP compared to the EV control (Fig 11E). We also examined the expression of plant defense responses related genes including the Pathogen-Associated Molecular Pattern-induced gene (*Cyp71D20*), Reactive Oxygen Species production related gene (*RBOHB*), and ethylene-responsive gene (*CTR1*) in response to the over-expression of SsHADV1 REP 48 hours post fungal inoculation. We observed an upregulation of those genes in the over-expressing SsHADV1 REP leaves compared to that of the EV control (Fig 12). Altogether the results suggest that the expression of SsHADV1-REP in plants limits fungal growth likely through repression of fungal aggressiveness in addition to priming plant immune system.

### Transient expression of SsHADV1 REP induces plant defense responses

**Fig 13:**
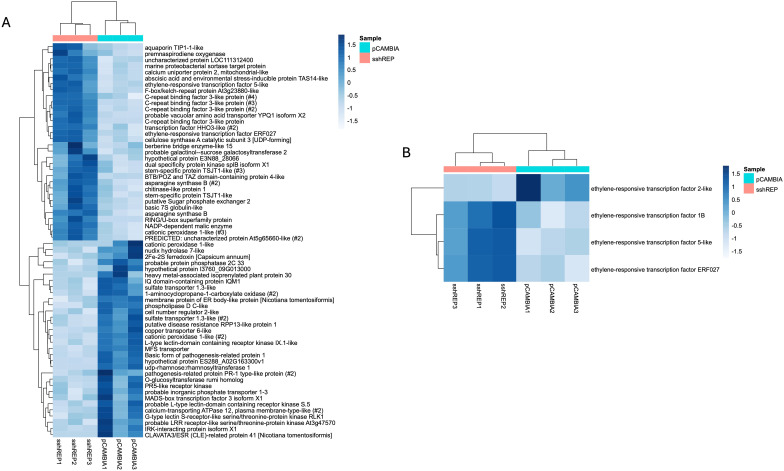
The Differential Gene Expression Analysis of *Nicotiana benthamiana* Transiently Expressing REP Shows that REP Induces Specific Responses. **(A)** Genes differentially expressed upon REP expression with a log2 fold change cutoff of 3.5; **(B)** Ethylene-responsive factors upregulated upon REP expression.

**Fig 14:**
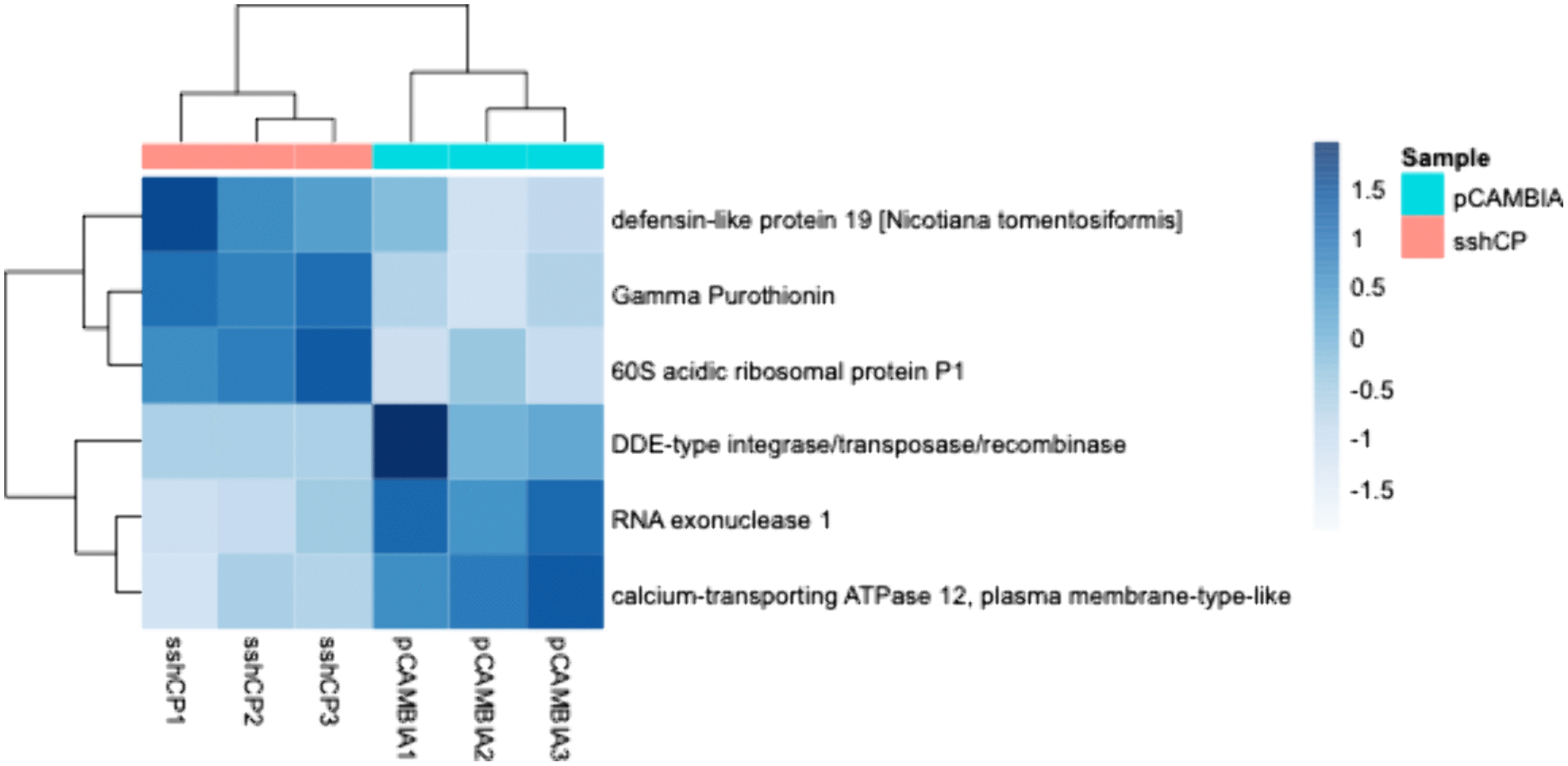
Differential Gene Expression Upon CP Transiently Expressed in *Nicotiana benthamiana.* Genes differentially expressed upon CP expression with a log2 fold change cutoff of 2.

To assess the overall changes in plant gene expression upon over-expression of SsHADV1 REP in plants, we performed RNA-Seq analysis on total RNAs extracted from *N. benthamiana* over-expressing SsHADV1 REP, SsHADV1 CP, and an empty vector (EV) as the control. We first validated the expression level of the SsHADV1 REP and CP transcripts (S3 Table). Upon transient expression of SsHADV1 REP, 196 genes were upregulated, and 160 genes were downregulated with the cutoff of log_2_ of 2 (>4-fold changes) when compared to the control EV. The list of the genes differentially expressed greater than 3.5-fold changes was summarized in Fig 13A including genes related to the ethylene signaling pathway such as ethylene-responsive transcription factor 1B (Nbe04g18970, Nbe06g37830), 5-like (Nbe04g32460), and ERF027 (Nbe11g12340), in line with transcriptome analysis done on plants inoculated with SsHADV1 infected *S. sclerotiorum* (Fig 13B). Many of the upregulated genes are involved in stress responses (Table 1). Gene ontology analysis of the biological processes enriched in the upregulated genes are related to urea transmembrane transporter activity, proteinogenic amino acid metabolic process, response to wounding, cellular response to lipid, response to water deprivation, cold, and abscisic acid, whereas inorganic anion transmembrane transport and cellular response to starvation are over-represented in the downregulated genes (Table 1). The expression of the SsHADV1 CP protein exerted less effect on gene differential expression, with only 6 genes differentially expressed after filtering the very low expression genes (0 read count) (Fig 14). Taken together, when expresssed in plants SsHADV1 REP shows a dual suppressive role in its hypovirulence activity: it disables the fungal pathogenicity factors, and it can prime plant defense responses.

**Table 1:** Gene ontology analysis of the biological processes upon SsHADV1 REP expression.

	GO biological process complete	Fold enrichment	GO
UP-Regulated genes	urea transport	>100	GO:0015840
	one-carbon compound transport	96.52	GO:0019755
	response to wounding	7.56	GO:0009611
	response to stress	2.54	GO:0006950
	response to stimulus	2.27	GO:0050896
	response to abscisic acid	5.08	GO:0009737
	response to lipid	4.54	GO:0033993
	response to chemical	2.91	GO:0042221
	response to hormone	3.27	GO:0009725
	response to endogenous stimulus	3.25	GO:0009719
	response to alcohol	5.01	GO:0097305
	response to oxygen-containing compound	4.15	GO:1901700
	response to abiotic stimulus	3.00	GO:0009628
	Unclassified	0.94	NA
Down-regulated genes	inorganic anion transmembrane transport	28.04	GO:0098661
	inorganic anion transport	15.87	GO:0015698
	inorganic ion transmembrane transport	9.56	GO:0098660
	Unclassified	1.31	NA

## Discussion

While hypovirulence associated mycoviruses are attractive as potential new sources of bio-fungicide, little is known about the viral-encoded protein responsible for the hypovirulence and their potential mode of action. Here, we identified the SsHADV1 replication associated protein or REP as the mycoviral anti-fungal agent of its fungal host *Sclerotinia sclerotiorum*. Using a combination of molecular and biological function analyses, we found that the SsHADV1 REP protein was sufficient to replicate fungal disease repression outside the context of full virus infection and is a potent regulator of fungal growth and aggressiveness.

First, we established that the expression of SsHADV1 REP limited fungal growth and disease development of both its natural host *S. sclerotiorum* but also its non-host *B. cinerea,* suggesting that SsHADV1 REP may harbor a broad-spectrum anti-fungal activity. The slow fungal growth was associated with the formation of hyphae bundles visible under the microscope with this characteristic linear aggregation of parallel-oriented hyphae, also known as mycelial cords. Mycelial chords are reported to form under environment conditions including nutrient depravation that requires the fungus to expand and explore new source of nutrients and to transport resources efficiently [21]. While the fungal response to SsHADV1 REP remains to be further studied, this is in line with a stress response.

Second, the repression of several *S. sclerotiorum* genes associated with fungal pathogenicity and growth by the SsHADV1 REP may affect not only the virulence of the fungus but also its ability to resist basal host defense responses, which could account for the hypovirulence phenotype observed in REP-over-expressing plants and in line with this interference of plant-fungal interaction and disease outcome. In fact, in the RNA-Seq analysis of *N. benthamiana* transiently expressing SsHADV1 REP or CP, and supported by the RT-qPCR, we found that plant defense related genes including the ethylene-responsive transcription factors (ERFs) were upregulated in response to REP expression and following fungal infection. ERFs are reported to regulate the expression of downstream stress-related genes and act either as activator or repressors and are often associated with fungal infection. The RT-qPCR analysis of additional plant defense response-related genes revealed their elevated activation upon fungal infection in the REP-overexpressing leaves. Altogether, our findings support that SsHADV1 REP serves thus a dual role. In addition to its direct activity on fungal growth and aggressiveness, it can prime the plant host defense responses. While it remains to be shown whether and how SsHADV1 REP can modulate plant host gene expression in the native context of fungal infection and knowing that the full SsHADV1 virus does not infect nor move in plant cells [15], several studies have established the cross-kingdom transmission of viruses from plant to fungus and insect [32,33] and their exchange of molecules including proteins via extra cellular vesicles [20,34]. It is worth noting that the PyAMPA prediction platform for antimicrobial peptide discovery and properties [10] indicated that REP protein has broad-spectrum activity across different microbes and despite a shorter half-life it is predicted to show lower toxicity (0.01) and decent cell-penetrating ability (0.61), which could possibly facilitate molecule exchange.

Third, SsHADV1 REP increases fungal sensitivity to the chemical boscalid and thus reducing the amount of fungicide needed to achieve the same level fungal growth inhibition as the effective concentration for 50% lethality. Boscalid works by inhibiting mitochondrial respiration in fungi and thus production of ATP [35]. While the mechanism of action and targets may be different, the boscalid and the SsHADV1 REP might likely act synergistically. It is clear though that the presence of the SsHADV1 REP did not increase fungal membrane permeability, as reported for some defensin peptides [20]. From a biological application perspective, boscalid is listed as a medium to high-risk fungicide for resistance development. SsHADV1 REP treatment could potentially delay the development of fungicide resistance by lowering fungicide application level in the fields.

Proper utilization of viral derived molecules requires a detailed understanding of their mode of function both in a native context and within the target organism. Replication initiator proteins like REP are essential proteins needed for DNA replication process [36]. They are crucial for the accurate duplication of the genome during cell division. Replication initiator proteins are known to interact with various host proteins to manipulate the plant’s cellular machinery and reprogram cell cycle, ensuring the virus can replicate and spread effectively [23]. For example, REP from Tomato yellow leaf curl virus interacts with 54 tomato proteins, including the EWS-like RNA-binding protein and THO complex subunit 4A [37]. The THO subunit 4 is part of the THO/TREX (TRanscription-EXport) complex, which controls RNA splicing and nuclear export of mRNA to the cytoplasm and is also connected to plant disease resistance [38]. Additionally, REP functions in reprograming of cell cycle to favor viral replication by releasing the block at G1/S phase. It interacts with retinoblastoma-related protein (RBR) via a conserved LXCXE motif and disrupts the E2F-RBR binding [23] that leads to activation of the expression of host DNA polymerases and other replication factors, which are important for viral replication and which are regulated by E2F transcription factors [23]. Previous transcriptome analysis of SsHADV1- infected fungus revealed upregulation of genes in pathways involved in cell cycle, DNA replication and DNA repair [14]. In this study, we showed that the putative LXCXE motifs found in SsHADV1 REP did not seem to have a function in hypovirulence. However, we established the importance of the ATPase domain of the REP for hypovirulence activity. While the predicated anti-microbial motif appears to be conserved within the REP sequences of several DNA viruses, we revealed protein structure may be important for the hypovirulence activity in addition to a functional ATPase domain. The striking structural similarity between the SsHADV1 REP and that of the SlaGemV1 REP despite their low sequence identity may explain their functional similarities, unlike the BcDV1 REP. It remains to be determined whether the REP function is also host-dependent as BcDV1 is a mycovirus of *B. cinerea* [29].

A novel aspect of this study is the biological application of SsHADV1 REP as an anti-fungal agent using an alternative delivery strategy that does not involve creating transgenic plants. This approach utilizes engineered plant growth-promoting rhizobacteria, which are environmentally friendly and non-pathogenic [39]. *B. subtilis* is one of the most extensively studied Gram-positive rhizobacteria, renowned for its remarkable protein secretion capabilities [40]. The advantages of *B. subtilis* in secreting proteins include high product yields, lack of toxic by-products, simplified downstream processing, and cost-effectiveness [41,42]. Recent advancements in plant pathology have seen a surge in research on the genetic engineering of *B. subtilis* to secrete specific compounds for disease control. For example, it has been engineered to secrete neuropeptides as novel nematicides, reducing tomato infection levels by up to 90% [43]; plant-defense elicitors like StPep1, enhancing potato resistance against root-knot nematodes [44]; and flg22, a conserved pathogen-associated molecular pattern (PAMP), to boost plant growth and immunity [45]. Our engineered *B. subtilis* to express and secrete mycovirus proteins demonstrated effectiveness in soil for at least 10 days and holds great potential for restoring fungal susceptibility to fungicides. These findings highlight the use of viruses as novel source of anti-fungal agents and that bacteria could serve as an effective delivery system, eliminating the need for transgenic plants. While it remains to be shown how the externally applied SsHADV1 REP is acquired by the fungus, several studies have established the cross-kingdom exchange of molecules including proteins via extra cellular vesicles [20,34], which could be a potential route for acquisition. In conclusion, the observation that mycoviruses and their encoded proteins can suppress fungal pathogen infection underlines their role as potential drivers of evolution of disease epidemics and severity.

## Materials and methods

### Construction of site-directed mutagenesis vectors and protoplast transformation of *Sclerotinia sclerotiorum*

The site-direct mutagenesis vectors for *S. sclerotiorum* were modified from pNDH-OGG and pNAN-OCT with GFP or mCherry fusion protein [16], which replaced with *S. sclerotiorum* nitrite reductase- or nitrate reductase-flaking regions, by using the In-fusion Snap assembly master mix (Takara; Fig 1A and S4 Table), called pOGG-Ss and pOCT-Ss. The nitrate assimilation genes, *nirD* and *NADPH* are not required for *S. sclerotiorum* growth and virulence on the cultural medium with other nitrogen sources [16]. The sequences of the plasmids were confirmed by whole plasmid sequencing (Genewiz, Azenta Life Sciences).

Protoplasts were prepared from *S. sclerotiorum* isolate 1980 as modified protocol of Chen et al. [46]. Virus-free *S. sclerotiorum* isolate 1980 plugs were first added and cultured in 100 ml YEPD for 4 days. The collected hyphae were washed once with dH_2_O and twice with the protoplast buffer (0.8 M MgSO·7H_2_O, 0.2 M C_6_H_5_Na_3_O_7_·2H_2_O, pH 5.5). 0.1 g hyphae was resuspended in 10 ml of enzyme solution (3 ml 1 M sorbitol, 50 mM sodium citrate, pH 5.8 with 100 mg lysing enzyme, 76.8 mg pectinase, and 236.8 mg glucanase and 1 mg chitinase/17 ml protoplasting buffer) at 90 rpm under 28^o^C for 3 hr. The protoplasts were filtered through 2 layers of miracloth and centrifuged at 3000x *g* for 5 min to pellet them. The protoplasts were then washed and resuspended with STC buffer (1 M sorbitol, 50 mM Tris-HCl, pH 8, 60 mM CaCl_2_) to approximately 1.3 × 10^6^ cells/100 μl STC for further transformation. The lineal pOCT-Ss/pOGG-Ss plasmids were cleaved with SacII and transformed into *S. sclerotiorum* by electroporation. The colonies were selected on the PDA plates with appropriate antibiotics, hygromycin (50 μg/ml, pOGG-Ss) or nourseothricin (50 μg/ml, pOCT-Ss) and transferred at least five times. To confirm the recombination, PCR was conducted using Plant direct kit with appropriate primer sets. For fluorescent microscopic observations, the transformants were cultured in PDB for 48 hr and observed by Olympus IX81.

### Site-specific integration of viral proteins expressed in *S. sclerotiorum*

In order to determine the function of each SsHADV1 protein in hypovirulence, the coding sequences of the SsHADV1-REP or -CP were separately cloned into pOGG-Ss, named as pOGG-Ss-SD-Rep, and pOCT-Ss, named as pOCT-Ss-SD-CP (Fig 2A and S4 Table). The sequences of the plasmids were confirmed by whole plasmid sequencing (Genewiz, Azenta Life Sciences). The protoplasts electroporation transformation as described previously and the transformants were chosen using hygromycin- or nourseothricin- containing PDA plates. and transferred at least five times. To confirm the recombination, PCR was conducted using Plant direct kit with appropriate primer sets (S4 Table). For fluorescent microscopic observations, the transformants were culture in PDB for 48 hr and observed by Olympus IX81. Transformants verified to be correctly expressing the genes at the specific gene-displacement sites were further compared for the virulence effect on *Nicotiana benthamiana* detached leaves.

### Growth rate assay and virulence assay on *N. benthamiana* of *S. sclerotiorum* transformants

For the growth rate evaluation, transformed and virus-free *S. sclerotiorum* were cultured on Difco Potato Dextrose Agar (PDA) medium (BD, Sparks, MD, USA) at room temperature for 2 days. The fungal plug (0.5-cm-diameter) was taken and placed with mycelium-side down on the center of new plates and cultured at room temperature for 2 days.

For the virulence assay, the 0.5-cm-diameter mycelial plugs were obtained from fresh transformed and virus-free *S. sclerotiorum* culture grown on PDA. Fully expanded upper leaves of *N. benthamiana* were detached and placed on two layers of paper towels in a plastic storage box. The plugs were then placed with mycelium-side down on the detached leaf surface. Images of the lesion were captured after 24 hr.

### Site-specific integration of viral proteins expressed in *B. cinerea*

Using the NotI site, the coding sequences of the REP or CP genes (accession number NC_013116.1) were separately cloned into pNDH-OGG [16], a vector containing a hygromycin resistance gene cassette, in order to determine the function of each SsHADV1 protein in hypovirulence. The system’s benefit is that the plasmids were created to take the place of the endogenous bcniaD gene, which was found to be unnecessary in *B. cinerea* under normal growth conditions. The protoplasts with the constructs underwent PEG transformation as previously mentioned [47], and the transformants were chosen using hygromycin-containing PDA plates. By using diagnostic PCR and RT-PCRs detailed in [16], homologous recombination was confirmed in the DNA and RNA extracted from the transformants. For fluorescent microscopic observations, the transformants were observed by Olympus IX81. Transformants verified to be correctly expressing the genes at the specific gene-displacement sites were further compared for morphology and virulence effects on tomatoes and grapes.

### Transformation of *B. subtilis*

The nucleotide sequences coding for the SsHADV1 REP and CP proteins (accession number NC_013116.1), the mutant REPs, the SlaGemV1 REP (accession number NC_028460) and the BcDV1 REP (accession number QJT73729) were synthesized by Twist Bioscience and cloned into the multi-cloning site of pBE-S vector (Takara) using HindIII and XbaI sites. Next, the signal peptide library was inserted using in-fusion and transformed *E. coli* DH5a. The sequences of the plasmids were confirmed by DNA sequencing (Functional Biosciences). The verified plasmids were introduced into *B. subtilis* strain RIK1285 (Takara). Colony PCR was performed to confirm transformation of *B. subtilis* RIK1285. The functional signal peptide (*bglC*) was selected using the plate assay described below.

### Bacterial protein extraction and western blot

SsHADV1 REP and CP fused with a 5’ end HA tag, were cloned into the pBE-S vector with the *bglC* signal peptide using the same HindIII and XbaI sites. The new constructs were transformed into *B. subtilis* RIK1285, and colony PCR was used for confirmation. RIK1285(REP), RIK1285(CP) and RIK1285(EV) were grown in 10 mL of LB for 23 hours at 37°C, 220 rpm. 1 mL of the bacterial culture was centrifuged, and the bacterial pellet was collected for protein extraction and treated with lysozyme. Another 5 mL of the bacterial culture was centrifuged, and the supernatant was collected and concentrated using Amicon Ultra-4 centrifugal filters (Millipore). Protein samples from both the pellet and supernatant were equally loaded into Mini-PROTEAN TGX Gels (BIO-RAD) and run at 100 volts for 70 minutes. The gel was then transferred to a PVDF membrane. The primary antibody was HA-Tag (C29F4) Rabbit mAb (1:1000, Cell Signaling Technology), and the secondary antibody was anti-Rabbit (1:10000). The membrane was treated with SuperSignal West Dura Substrate (Thermo Scientific) and imaged using camera exposure. This experiment was repeated twice with consistent results.

### Fungal plate assay

Transformed *B. subtilis* was cultured at 37°C until the OD600 reached 2.5 (~23 hours). The bacterial culture was then centrifuged, and the supernatant was filtered through a 0.2 µm pore filter and supplemented with Protease Inhibitor Cocktail VII (DOT Scientific). Equal volumes of the bacterial cell filtrate were added to PDA plates containing spectinomycin (50 µg/mL) to eliminate any remaining bacteria, followed by the addition of a 2 mm actively growing *S. sclerotiorum* agar plug, placed with mycelium-side down on the center of PDA plates. Images of the mycelium were captured at 24 and 48 hours after inverting the plug on the surface of the PDA, where the mycelium diameter was measured at five different angles. For experiments extending beyond 48 hours, an additional dose of cell filtrate was added at 48 hours after inverting the mycelial plug. Each experiment was performed in triplicates and in three independent repeats.

### Sunflower plant assay

*B. subtilis* cultures (OD600 = 0.6) expressing specific proteins were resuspended in equal amounts of water. Sunflowers RHA373 [48] at the V6 stage were soaked in 500 mL of the bacterial suspension via soil for 2 hours. Two days later, an additional 10 mL of the bacterial suspension was applied to each sunflower. After another two days, a 2.5-cm diameter *S. sclerotiorum* (1980) agar plug was inoculated beneath the root mass, in direct contact with the roots. The stem lesion size was measured using imageJ, photographs of root condition and the overall growth of the sunflowers were recorded for up to 8 days post-fungal inoculation. The experiment was performed with a minimum of 3 to maximum of 11 plants per treatment with three independent repeats.

### Oxalic acid level measurement

A standard plate assay was conducted with *S. sclerotiorum* (1980) using PDA plates containing spectinomycin (50 µg/mL). At 48 hours post-infestation (hpi), three same-sized agar plugs from each treatment (taken from the outer edge of the mycelial mat, avoiding the hyphae) were collected using the back of a 200 µL pipette tip (5mm). Each plug was placed in a 1.5 mL tube containing 500 µL of nuclease-free water and incubated at 25°C with shaking at 500 rpm for 90 minutes. The solution was then collected as a sample for analysis using the Oxalate Assay Kit according to the manufacturer’s instructions (Sigma-Aldrich). The experiment was performed in triplicate with two independent repeats.

### Fungicide treatment

A standard plate assay was conducted at room temperature with 2 mm *S. sclerotiorum* (1980) agar plug using PDA plates containing spectinomycin (50 µg/mL), amended with the commercial boscalid fungicide (succinate-dehydrogenase inhibitor; Fungicide Resistance Action Committee group 7) at final concentrations of 0.1 ppm and 0.01 ppm resuspended in acetone [35]. PDA medium amended with 0.01% (vol/vol) acetone was used as the non-treated control.

### Hyphal Imaging and Plasma membrane permeabilization assay

At 48 hours post-infestation, hyphal images were taken under bright field using a BioTek Cytation 7. Images were next processed by Image J for measurement of the hyphal bundle width.

Membrane permeabilization of fungal cells was analyzed using a BioTek Cytation 7 imaging reader by visualizing the influx of SYTOX Green Nucleic Acid Stain (Thermo Fisher Scientific, USA). A standard plate assay was conducted with *S. sclerotiorum* (1980) grown on PDA plates supplemented with spectinomycin (50 µg/mL) followed with the different cell filtrate treatments. 48 hours post incubation, 1 μM SYTOX Green (diluted 1:5000 in deionized water) was added to fully cover the fungal mycelium. The plates were incubated in the dark for 30 minutes. SYTOX Green was then removed, and the plates were washed three times with deionized water. The 100 mm Petri dishes were imaged using a 465 nm LED light source and a filter set (excitation: 469/35 nm; emission: 525/39 nm). *S. sclerotiorum* plates exposed to UV light for 10 minutes at 254 nm (Stratalinker UV crosslinker 1800) served as a positive control. Images were processed using Gen5 3.13 software.

### Antimicrobial sequence scanning

PyAMPA was run on Google Colab [49], a new computational prediction platform, for antimicrobial peptide discovery and properties [24]. Using default settings, SsHADV1 REP [50] and CP [51] were predicted with antimicrobial peptides from the entire proteomes, providing results that included antimicrobial motifs, toxicity, half-life, and other relevant properties.

### Protein transient expression in plants

Four weeks-old *N. benthamiana* leaves were infiltrated with agrobacteria GV3101 (OD600 = 0.8) expressing SsHADV1 REP, SsHADV1 CP, and an empty vector (EV) control. At 48 hours post infiltration, three leaves of each treatment were exposed to an actively growing 2mm agar plug of wild-type (WT) *S. sclerotiorum* (isolate 1980) cultured on PDA (Difco) plates, incubated at room temperature. The extent of leaf necrosis was recorded up to 48 hours post inoculation (hpi) and measured by image J. The experiment was repeated at least three times*.*

### Gene-expression analysis by RT–qPCR

At 48 hpi, 100 mg of leaf tissue from both necrotic and healthy leaf areas was collected for total RNA extraction using the RNeasy plant mini kit (Qiagen). Each leaf was treated as an individual biological replicate; three biological replicates were collected for each sample treatment. The cDNA was synthesized with iScript Reverse Transcription Supermix for RT-qPCR kit (BIO-RAD). RT-qPCR was performed using SsoFast EvaGreen Supermixes (BIO-RAD) and analyzed using the housekeeping gene *S. sclerotiorum β-tubulin*. The experiment was repeated at least three times. The primer sequences are listed in S5 Table.

### RNAseq analysis

At 48 hours post infiltration prior to *S. sclerotiorum* inoculation, leaves infiltrated to express REP or CP were collected and RNA was extracted using the RNeasy plant mini kit (Qiagen). RNA-Seq libraries were built by sending total RNA extracts to Novogene (Beijing, China) for sequencing. Illumina TruSeq RNA library kit was used on the RNAs extracted from the three individual *N. benthamiana* plants with the leaves infiltrated by a binary vector, pCambia1302, expressing REP or CP without GFP.

Adaptor trimming of raw reads was completed with BBDuk [52], allowing for one mismatch. Quality of the reads was checked through FastQC [53]. HISAT2 [54] was used for genome alignment, utilizing the *N. benthamiana* genome assembly NbeHZ1_genome_1.0 accessed through http://lifenglab.hzau.edu.cn/Nicomics/index.php [55]. File conversions were done through samtools [56] to sort the bam output files and feature Counts [57] was used to output a gene feature count text file for differential expression analysis. Differential gene expression analysis was done with the R statistical suite with packages “DESeq2” for normalization and differential expression, and “apeglm” for its shrinking algorithms for visualization [58–60]. pCAMBIA1302 was used as the reference data set for expression analysis and expression was determined as log_2_ fold changes. A protein database was built from the NbeHZ1 1.0 genome and annotation file with gffread [61] to create descriptions for individual genes with the BLAST function of Blast2Go [62]. Differentially expressed genes with a of log_2_ cutoff of 2 were converted from NbeHZ1 (*N. benthamiana*) to TAIR11 (*Arabidopsis thaliana*) homologues for GO enrichment by the TAIR GO Term Enrichment for Plants tool utilizing PANTHER [63–65].

The PCA biplot was constructed with R packages “DESeq2” and visualized with “ggplot2” [66]. Gene expression heatmaps were created with the R package “pheatmap” [67] for comparisons between empty pCAMBIA1302 and pCAMBIA1302 + REP or pCAMBIA1302 + CP. Ethylene-responsive transcription factor-like and abscisic acid-associated genes were identified by their BLAST descriptions and lists were filtered and analyzed separately. Data have been archived in the NCBI under BioProject Accession number: PRJNA1297152

### Statistical analysis

Statistical analyses were conducted using R software and Excel. Data normality was assessed using the Shapiro-Wilk test, and homogeneity of variances was evaluated with Levene’s test. For data that met assumptions of normality and equal variances, significance was determined using one-way analysis of variance (ANOVA), followed by Tukey’s Honest Significant Difference (TukeyHSD) test. For comparisons between two groups, a one-tailed, two-sample equal variance (homoscedastic) t-test was applied unless otherwise specified. Statistical significance is indicated as “*” for *P* < 0.05 and “**” for *P* < 0.01. Error bars represent the standard error of the mean (SEM) based on at least three replicates.

## Supporting information

S1 Fig Confirmation of Site-Directed Expression of REP and CP in *B. cinerea.* (A) The gene displacement was validated for transformants BcVB-CP-Ss and BcVB-REP-Ss using the diagnostic PCR primers (bcniaD-hi5F/TgluchiF, hph-hiF/bcniaD-hi3R). Lane 5’: Confirmation of the size of the 5’ region of homologous integration transformants; Lane 3’: confirmation of the 3’ region. Lane M: ladder; Lane NC: BcVB-OGG transformant of the empty vector of OGG. (B) Confirmation of the REP and CP gene overexpression by RT-qPCR. BcVB, wild-type *B. cinerea*; BcVB-OGG, BcVB transformed by empty OGG vector; BcVB-CP-Ss, CP transformant; BcVB-REP-Ss, REP transformant.
(TIF)

S2 Fig Quantification of *B. subtilis* Counts for RIK1285(EV), RIK1285(CP), and RIK1285(REP) in Soil Post-Fungal Infection. Bacterial cell counts for RIK1285(EV), RIK1285(CP), and RIK1285(REP) were measured in sunflower soil samples collected at 0, 4, and 8 dpi using selective LB plates containing cycloheximide (100 µg/mL) and kanamycin (10 µg/mL) [50]. Statistical analysis of the bacterial populations at each time point is provided.
(TIF)

S3 Fig Relative expression level of the oxalic acid production-related gene (Ssoah) in RIK1285(EV) and RIK1285(REP) treated *S. sclerotiorum* on plates. Mycelium growing on PDA plates with cellophane containing spectinomycin was collected for total RNA extraction 48 hours post-fungal inoculation, followed by RT-qPCR analysis using the housekeeping gene *β-tubulin*.
(TIF)

S4 Fig Membrane permeabilization activity and SYTOX Green uptake in fungal cells treated with mycoviral proteins. Fluorescence and corresponding bright-field microscopy images showing SYTOX Green uptake in *S. sclerotiorum* 1980 hyphae treated with the supernatants of RIK1285(EV), RIK1285(CP), and RIK1285(REP). *S. sclerotiorum* exposed to UV light for 10 minutes at 254 nm served as the positive control, and wild-type *S. sclerotiorum* as the negative control. Scale bar = 200 μm.
(TIF)

S5 Fig Putative LXCXE-Like Motifs in SsHADV1 REP Are Not Required for Hypovirulence Activity. Equal volumes of the *B. subtilis* cell filtrate from RIK1285(EV), RIK1285(REP), RIK1285(REP-LGCQ), RIK1285(REP-LHCF) and RIK1285(REP-LGCQ+LHCF) were added to PDA plates containing spectinomycin, followed by the addition of an *S. sclerotiorum* agar plug. Statistical analysis of the mycelium diameter at 24 and 48hpi is presented. Error bars represent the SEM. Statistical significance analyzed using a one-tailed t-test, is indicated as follows: “*” for *P* < 0.05 and “**” for *P* < 0.01.
(TIF)

S1 Table Numbers of healthy and dead sunflowers following *S. sclerotiorum* infection after combined independent treatments with bacterial cultures secreting SsHADV1 REP, CP, or empty vector (EV). (XLSX)

S2 Table Antimicrobial motif and activity prediction of SsHADV1 REP using PyAMPA. (XLSX)

S3 Table Read counts for the SsHADV1 REP and CP transcripts in *N. benthamiana* overexpressing SsHADV1 REP, SsHADV1 CP, and the empty vector PCAMBIA, relative to the total reads. (XLSX)

S4 Table Primer sequences used for In-fusion cloning and PCR confirmation of *S. sclerotiorum* transformants. (XLSX)

S5 Table (XLSX)
